# Publisher Correction: Optimal compressed representation of high throughput sequence data via light assembly

**DOI:** 10.1038/s41467-018-03711-0

**Published:** 2018-04-26

**Authors:** Antonio A. Ginart, Joseph Hui, Kaiyuan Zhu, Ibrahim Numanagić, Thomas A. Courtade, S. Cenk Sahinalp, David N. Tse

**Affiliations:** 1 Department of Electrical Engineering, Stanford University, Stanford, CA 94305 USA; 2 Department of Electrical Engineering & Computer Science, Massachusetts Institute of Technology, Cambridge, MA 02139 USA; 3 Department of Computer Science, Indiana University Bloomington, Bloomington, IN 47405 USA; 4 Computer Science & Artificial Intelligence Laboratory, Massachusetts Institute of Technology, Cambridge, MA 02139 USA; 5 Department of Electrical Engineering and Computer Sciences, University of California, Berkeley, CA 94720 USA

Correction to: *Nature Communications*, 10.1038/s41467-017-02480-6, published online 08 February 2018

The original version of this Article contained errors in the affiliations of the authors Ibrahim Numanagić and Thomas A. Courtade, which were incorrectly given as ‘Department of Electrical Engineering and Computer Sciences, University of California, Berkeley, CA 94720, USA’ and ‘Computer Science & Artificial Intelligence Laboratory, Massachusetts Institute of Technology, Cambridge, MA 02139, USA’, respectively. Also, the hyperlink for the source code in the Data Availability section was incorrectly given as https://github.iu.edu/kzhu/assembltrie, which links to a page that is not publicly accessible. The source code is publicly accessible at https://github.com/kyzhu/assembltrie. Furthermore, in the PDF version of the Article, the right-hand side of Figure 3 was inadvertently cropped. These errors have now been corrected in both the PDF and HTML versions of the Article.

